# Clinically oriented automatic 2D liver tumor segmentation: LCMambaNet with a state-space model and liver cancer–specific attention

**DOI:** 10.3389/fonc.2026.1676424

**Published:** 2026-02-03

**Authors:** Pengcheng Sun, Jing Yu, Qi Gu, Luping Zhang, Yuhan Sun, Qin Wang, Liugen Gu, Jianchun Zhu

**Affiliations:** 1 Department of Interventional Radiology, Suzhou Xiangcheng People’s Hospital, Suzhou, China; 2 Department of Gastroenterology, the Southeast University Affiliated Nantong First People's Hospital, Nantong, China; 3 Department of Gastroenterology, the First People’s Hospital of Nantong, Nantong, China; 4 School of Medicine, Nantong University, Nantong, Jiangsu, China; 5 Affiliated Nantong Hospital 3 of Nantong University, Nantong, China; 6 Department of Radiology, Suzhou Xiangcheng People's Hospital, Suzhou, China

**Keywords:** 2D efficient networks, attention mechanism, liver cancer segmentation, medical imaging, state space models

## Abstract

**Introduction:**

Liver cancer is among the deadliest malignancies worldwide, and both its incidence and mortality continue to rise. Precise tumor segmentation often remains difficult due to heterogeneous enhancement patterns, infiltrative margins, and frequently obscured underlying parenchymal disease. While deep learning has advanced the field, existing heavy 3D architectures (e.g., nnU-Net) often require substantial computational resources, which limits their clinical deployment. Standard architectures also still struggle to reconcile fine-grained tissue cues with whole-organ context.

**Methods:**

This study introduces the Liver Cancer Mamba Network (LCMambaNet), an efficient 2D segmentation framework built on selective state-space models. A tailored scan-patch mechanism extracts salient texture- and density-based features, sharpening the discrimination between normal parenchyma and malignant regions. The Liver Cancer Attention Module (LCAM) further decouples the confounding relationships between parenchymal descriptors and tumor characteristics. The selective state-space backbone captures long-range dependencies and continuous feature dynamics. We evaluated the model on both the LITS (CT) and CirrMR160+ (MRI) datasets.

**Results:**

The proposed approach surpasses current state-of-the-art methods, achieving Dice scores of 92.94 ± 3.12% and 92.08 ± 2.85% on the LITS and CirrMR160+ datasets, respectively. Notably, stratified analysis shows superior performance on small lesions (< 2 cm), with statistical significance (p < 0.01) against strong baseline models. Comprehensive ablation studies verify the contribution of each component.

**Discussion:**

The results demonstrate that LCMambaNet offers an efficient, clinically viable solution for 2D liver tumor segmentation. Its design addresses the key limitations of existing models, balancing computational efficiency with high segmentation accuracy. The strong performance on small lesions also highlights its potential to support early diagnosis and precise treatment planning, advancing the clinical utility of AI-based segmentation tools.

## 1 Introduction

Hepatocellular carcinoma (HCC) is among the most lethal cancers worldwide, with incidence and mortality continuing to rise Jiang et al. (1) Polat et al. (2) Siegel et al. (3). As the fourth leading cause of cancer-related death, it remains difficult to detect and treat early Jesi and Daniel (4). HCC typically arises on a background of chronic liver disease—viral hepatitis, alcoholic liver disease, or non-alcoholic fatty liver disease—with cirrhosis as the strongest risk factor Emam et al. (5) Li et al. (6). Even with modern imaging, early detection is impeded by heterogeneous appearance, infiltrative growth, and the complex milieu of chronically diseased parenchyma Tejaswi and Rachapudi (7).

Current State-of-the-Art (SOTA) in medical segmentation is dominated by 3D volumetric models. The self-configuring nnU-Net? and Transformer-based architectures like UNETR? and Swin-UNETR? have set high benchmarks. However, these 3D models incur high memory costs and latency, posing challenges for real-time clinical workflows Xing et al. (8). Conversely, 2D approaches are efficient but traditionally lack global context. Despite notable gains in liver segmentation Chen et al. (9), liver cancer segmentation remains difficult due to: (1) phase-dependent variability in HCC enhancement Archana and Anand (10), (2) benign lesions that mimic malignancy Wu et al. (11), (3) intratumoral heterogeneity with necrosis Gul et al. (12), and (4) architectural distortion from cirrhosis Zhang et al. (13)Vijayaprabakaran et al. (14).

Transformers have advanced sequence modeling by using self-attention to capture long-range dependencies without recurrence. However, the quadratic scaling of self-attention with sequence length limits their efficiency on long inputs Li et al. (15). To mitigate this, recent work integrates State Space Models (SSMs) Zhou et al. (16) Wang et al. (17) Liu et al. (18) Ma et al. (19) Ruan et al. (20) into Transformer-like designs, yielding Mamba-style architectures that replace self-attention with linear recurrent layers derived from state-space formulations Wang et al. (21) Liao et al. (22) Liu et al. (23).

Furthermore, recent advances have explored incorporating domain-specific constraints and discrete representation learning to improve segmentation robustness. Approaches utilizing anatomical priors Lastname and Others (24) guide the network using shape constraints, while Vector Quantized Variational Autoencoders (VQ-VAE) employing codebook-based learning Lastname and Others (25) have shown promise in modeling discrete feature distributions to handle tissue heterogeneity. While effective, these methods often add complexity to the inference pipeline. In contrast, our approach seeks to balance representation power with inference efficiency.

**Figure 1:**
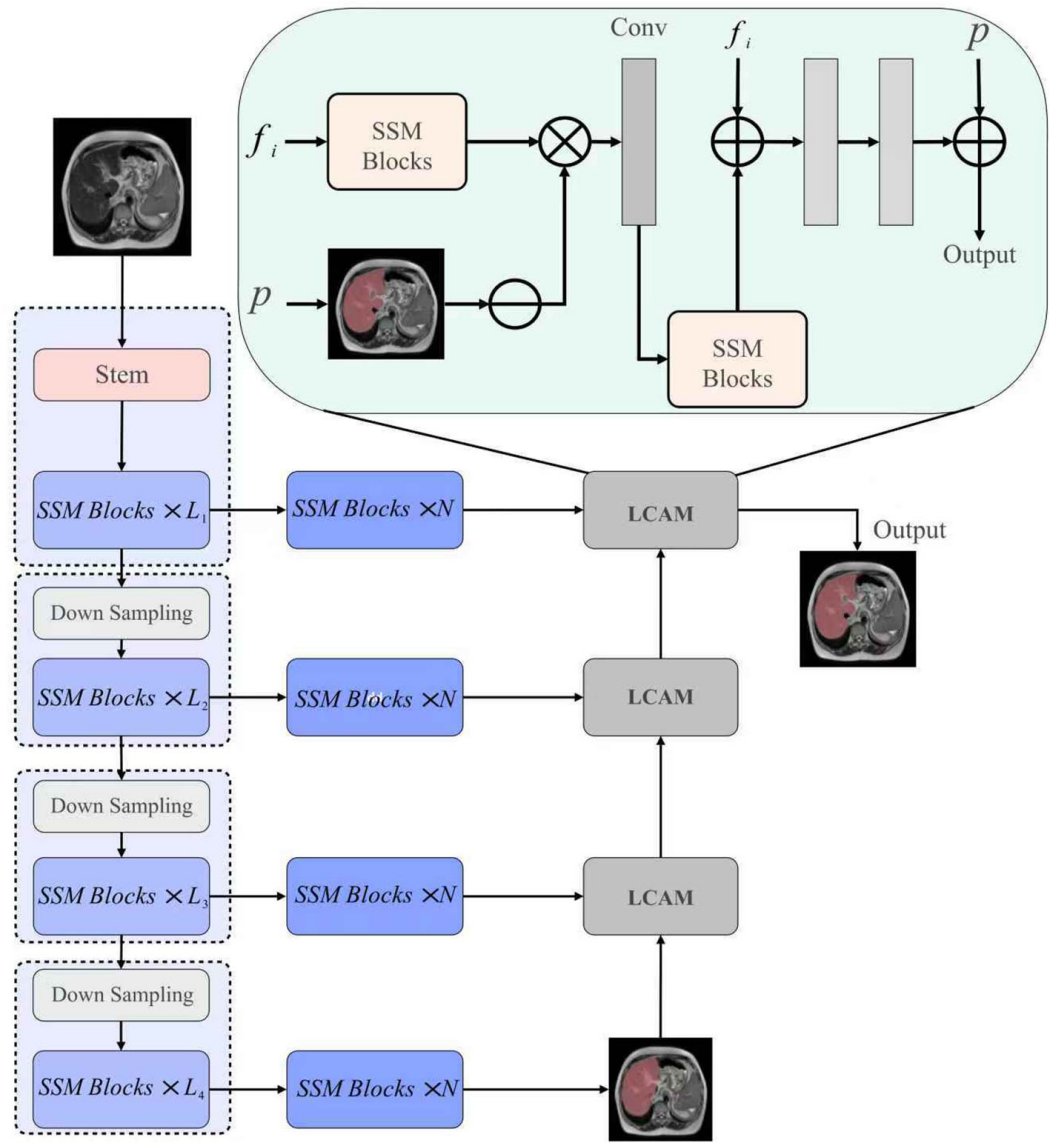
Overview of the proposed LCMambaNet architecture. The network processes 2D slices to ensure low-latency inference, utilizing a hierarchical Mamba encoder (Left) and the LCAM module (Right) to capture global context usually lost in 2D methods.

This work proposes the 2D Liver Cancer Mamba Network (LCMambaNet), a Mamba-based framework tailored for slice-wise liver cancer segmentation, as illustrated in **Figure 1**. We explicitly adopt a 2D strategy to maximize computational efficiency while leveraging Mamba’s ability to model long-range dependencies across the entire slice plane. The model learns factorized local–global representations, mines correlations between healthy parenchyma and tumor regions, and delivers an effective automated solution.

In summary, the contributions are:

1. Introduction of LCMambaNet, which combines a tailored scanning strategy, custom kernel operators, and selective state-space blocks to achieve accurate and efficient liver cancer segmentation.
2. Design of a liver-specific feature extractor to harvest critical tissue attributes from texture and density cues.
3. Development of a specialized SSM block that captures long-range dependencies, harmonizes local detail with whole-organ context.
4. Comprehensive experiments demonstrating state-of-the-art performance on public liver cancer datasets, including lesion-size stratification and statistical significance testing.

## 2 Method

### 2.1 Overview of the architecture

The proposed LCMamba Net architecture addresses the unique challenges of liver cancer segmentation through a meticulously designed hierarchical framework that leverages selective state space models. **Figure 1** illustrates the overall architecture, which comprises three key components:

1. A Mamba-based encoder that extracts multi-scale features.
2. Enhanced State Space Model (SSM) blocks for feature refinement.
3. A specialized Liver Cancer Attention Module (LCAM).

The encoder processes an input liver image slice $I\in {\mathbb{R}}^{H\times W\times 3}$ and extracts hierarchical features $f_{i}{i=}_{1}^{4}$. This 2D formulation significantly reduces the parameter count compared to 3D counterparts like V-Net or Swin-UNETR.

To enhance model flexibility while maintaining computational efficiency, two variants are considered:

- LCMamba-T: Utilizes Mamba-Tiny backbone with no additional SSM blocks (*N* = 0).
- LCMamba-S: Employs Mamba-Small backbone with one additional SSM block (*N* = 1).

The computational complexity of our model scales linearly with image size, as shown in Equation 1:

(1) $$O(\text{LCMamba}\;\text{Net})=O(HW)\cdot O(C^{2})\cdot O(N)$$

where *H*, *W* represent image dimensions, *C* denotes the maximum channel size, and *N* indicates the number of SSM blocks.

### 2.2 Encoder

The Mamba-based encoder forms the backbone of our architecture. The encoder processes input through a series of hierarchical stages:

1. Initial Embedding: A stem module transforms the input image into initial feature maps via a convolutional layer, as defined in Equation 2:

(2) $$f_{stem}={\text{Conv}}_{3\times 3}(I)$$

2. Multi-Stage Processing: The embedded features progressively pass through four stages, each containing SSM blocks, as described in Equation 3:

(3) $$f_{i}={\text{Stage}}_{i}(f_{i-1})$$

where each stage downsamples the spatial resolution while increasing channel dimensions, shown in Equation 4:

(4) $${\text{Stage}}_{i}:{\mathbb{R}}^{H_{i-1}\times W_{i-1}\times C_{i-1}}\to {\mathbb{R}}^{H_{i}\times W_{i}\times C_{i}}$$

### 2.3 State space model block

The core innovation in our architecture lies in the application of selective state space models for medical image processing. The SSM block implements a 2D-Selective-Scan module (SS2D) that efficiently models bidirectional dependencies across the image Zhu et al. (26). For an input feature map $X\in {\mathbb{R}}^{H\times W\times C}$, the SS2D operates along four scanning directions: →, ←, ↓, ↑. For each direction, the selective scan process is formulated as (Equation 5):

(5) $$\begin{array}{l} {\bar{h}}_{t}=\bar{A}h_{t-1}+\bar{B}x_{t}\\ y_{t}=\bar{C}h_{t} \end{array}$$

where $h_{t}\in {\mathbb{R}}^{N\times D}$ represents the hidden state at position *t*, *x_t_* is the input at position *t*. The learnable parameters are derived through discretization, as shown in Equation 6:

(6) $$\bar{A}=\text{exp}(\text{Δ}\cdot A),\bar{B}={\left(\text{Δ}\cdot A\right)}^{-1}(\text{exp}(\text{Δ}\cdot A)-I)\cdot \text{Δ}\cdot B$$

Here, Δ represents the discretization step size. The parameter *A* is structured to be selective, as defined in Equation 7:

(7) A=diag(Λ)

The outputs from the four scanning paths are combined to form a comprehensive representation, computed via Equation 8:

(8) $$Y=\text{Proj}(\text{Concat}[Y_{\to },Y_{\leftarrow },Y_{↓},Y_{↑}])$$

### 2.4 Liver cancer attention module

To directly tackle liver cancer delineation, we present LCAM to exploit multi-scale features to sharpen segmentation boundaries. Given an initial segmentation $P_{i+1}$ and an encoder feature map $f_{i}$, the module performs refinement. LCAM first derives an attention map $A_{i}=\text{Θ}(P_{i+1})$ with Θ(P)=1−P+γ·∇P. The attention then modulates encoder features via $R_{i}=A_{i}⊙\delta (f_{i})$. The overall operation is $\text{LCAM}(f_{i},P_{i+1})=p_{i}+\text{Upsample}(P_{i+1})$, combining refined features with the upsampled prior.

### 2.5 Loss function and optimization

The loss function is carefully designed to address the inherent class imbalance. We employ a weighted combination of Binary Cross-Entropy (BCE) and Dice losses, as formulated in Equation 9:

(9) $$L=\alpha L_{\text{BCE}}+\beta L_{\text{Dice}}$$

To specifically enhance performance at tumor boundaries, we introduce a boundary-aware term, shown in Equation 10:

(10) $$L_{\text{Boundary}}=1-\frac{2\sum_{i\in B}y_{i}{\hat{y}}_{i}+Є}{\sum_{i\in B}y_{i}+\sum_{i\in B}{\hat{y}}_{i}+Є}$$

The final loss function is the weighted sum of these components, as given in Equation 11:

(11) $$L=\alpha L_{\text{BCE}}+\beta L_{\text{Dice}}+\lambda L_{\text{Boundary}}$$

## 3 Experiments and results

### 3.1 Datasets

1) LiTS Dataset: The Liver Tumor Segmentation (LiTS) dataset comprises 201 abdominal CT scans. To ensure reproducibility and Evaluation Protocol Transparency, we utilized the official training set (130 scans) and performed a Fixed Internal Split (Seed=42): 100 scans for training, 15 for validation, and 15 for testing. We report metrics on this held-out test set.

2) CirrMRI600+ Dataset: This dataset includes 628 high-resolution abdominal MRI volumes from 339 patients. We followed the dataset’s predefined partitioning scheme.

### 3.2 Implementation and reproducibility

All experiments were implemented in PyTorch 1.13.0 with CUDA 11.7 and cuDNN 8.5 on a single NVIDIA RTX A10 GPU (24GB). To ensure reproducibility, random seeds were fixed to 3407. Encoders were initialized with pre-trained Mamba weights.

During training, images were resized to 256×256. We applied random rotation (± 15°), horizontal flips, and vertical flips. We used the Adam optimizer (initial LR 1 × 10^−4^) with ReduceLROnPlateau. Batch size was 16.

### 3.3 Evaluation metrics

We report Dice coefficient (Dice), mean Intersection over Union (mIoU), recall, precision, F2 score, and 95% Hausdorff distance (HD95). Following statistical rigor guidelines, all results are reported as Mean ± Standard Deviation (SD). Furthermore, we report the 95% Confidence Intervals (CI) for the primary Dice metric to quantify estimation uncertainty. To control the family-wise error rate (FWER) during multiple hypothesis testing across different models, we applied the Holm-Bonferroni correction (*p <* 0.05 considered significant).

### 3.4 Benchmarking

1) Results on CirrMRI600+ Dataset: Extensive experiments show that LCMamba Net achieves superior performance, as detailed in **Table 1**. Statistical significance was assessed using a paired Wilcoxon signed rank test. LCMamba-S shows significant improvement over TransResUNet (*p <* 0.01).

**Table 1:** Model performance on the CirrMRI600+ dataset (Mean ± SD).

Method	Dice (%)	95% CI	mIoU (%)	Recall (%)	Precision (%)	F2 (%)	HD95 (mm)
U-Net	89.40 ± 3.25	[88.2, 90.6]	84.10 ± 4.12	90.95 ± 3.88	91.66 ± 3.55	90.01 ± 3.62	3.52 ± 1.25
UNeXt	83.83 ± 4.50	[82.1, 85.5]	76.88 ± 5.20	85.57 ± 4.90	88.46 ± 4.10	84.46 ± 4.65	4.03 ± 1.88
TransNetR	90.35 ± 3.10	[89.2, 91.5]	85.05 ± 3.85	91.51 ± 3.60	92.10 ± 3.40	90.78 ± 3.45	3.50 ± 1.15
TransResUNet	91.47 ± 2.95	[90.4, 92.5]	86.52 ± 3.50	92.70 ± 3.20	92.60 ± 3.10	91.95 ± 3.15	3.42 ± 1.05
VM-UNet	90.34 ± 3.05	[89.2, 91.4]	85.08 ± 3.80	92.31 ± 3.55	91.51 ± 3.45	91.18 ± 3.52	3.52 ± 1.18
LCMamba-T	91.17 ± 2.98	[90.1, 92.2]	86.16 ± 3.65	92.34 ± 3.45	92.48 ± 3.30	91.65 ± 3.35	3.50 ± 1.10
LCMamba-S	**92.08 ± 2.85**	[91.1, 93.1]	**87.36 ± 3.40**	**92.96 ± 3.15**	**93.30 ± 3.05**	**92.42 ± 3.20**	**3.39 ± 0.95**

The 95% confidence interval (CI) is reported for the primary Dice metric.

For metrics where higher values indicate superior segmentation accuracy (Dice (%), mIoU (%), Recall (%), Precision (%), F2 (%)), the bold value is the maximum in the column.

For metrics where smaller values reflect more precise boundary delineation (HD95 (mm)), or lower computational cost (Params (M), FLOPs (G), Inference Time), the bold value is the minimum in the column. This notation intuitively highlights the core advantages of the proposed model/strategy.

**Figure 2:**
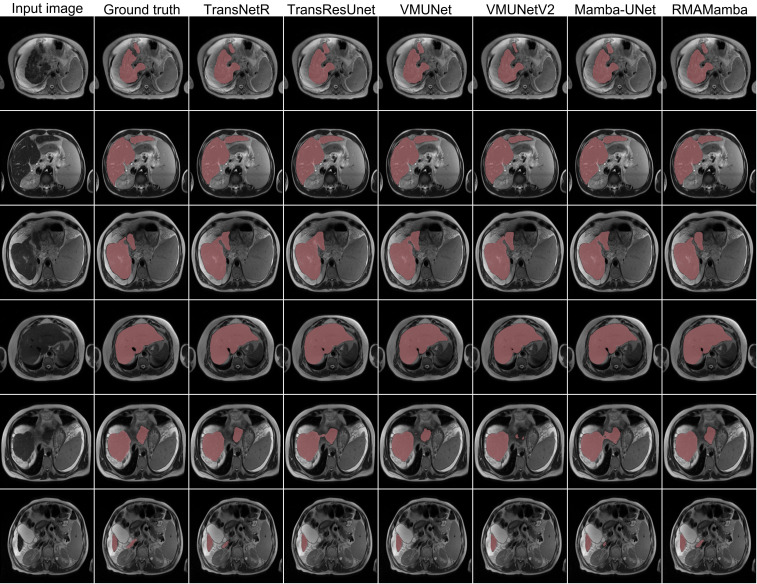
Qualitative results of different methods on the LiTS dataset.

Qualitative analysis through visual comparison, shown in **Figure 2**, further validates effectiveness on the LiTS dataset. Despite being a 2D method, LCMambaNet approximates the boundary delineation quality of 3D baselines while operating at significantly lower latency.

2) Results on LiTS Dataset: **Table 2** presents a comprehensive quantitative analysis on the Fixed Internal Split. Results are reported as Mean ± SD across the test cases. LCMamba-T attained the highest Dice coefficient of 92.94 ± 3.12%.

**Table 2:** Model performance on the LiTS dataset (fixed internal split).

Method	Dice (%)	95% CI	mIoU (%)	Recall (%)	Precision (%)	F2 (%)	HD95 (mm)
U-Net	90.79 ± 3.85	[89.3, 92.2]	86.31 ± 4.55	92.10 ± 4.10	92.49 ± 3.90	91.37 ± 4.05	2.97 ± 1.45
TransResUNet	92.58 ± 3.20	[91.4, 93.7]	88.42 ± 3.95	92.41 ± 3.65	95.01 ± 3.30	92.34 ± 3.55	2.86 ± 1.20
VM-UNetV2	92.05 ± 3.35	[90.8, 93.3]	87.95 ± 4.05	91.98 ± 3.75	94.86 ± 3.45	91.84 ± 3.68	2.87 ± 1.25
Mamba-UNet	91.88 ± 3.40	[90.6, 93.1]	87.77 ± 4.10	92.17 ± 3.80	94.42 ± 3.50	91.82 ± 3.70	2.89 ± 1.28
LCMamba-T	**92.94 ± 3.12**	[91.8, 94.1]	**88.99 ± 3.82**	92.34 ± 3.50	**95.59 ± 3.15**	92.44 ± 3.45	**2.83 ± 1.10**
LCMamba-S	92.74 ± 3.18	[91.5, 93.9]	88.92 ± 3.88	**92.91 ± 3.55**	94.92 ± 3.25	**92.67 ± 3.48**	2.86 ± 1.15

The 95% CI is included for the Dice score.

For metrics where higher values indicate superior segmentation accuracy (Dice (%), mIoU (%), Recall (%), Precision (%), F2 (%)), the bold value is the maximum in the column.

For metrics where smaller values reflect more precise boundary delineation (HD95 (mm)), or lower computational cost (Params (M), FLOPs (G), Inference Time), the bold value is the minimum in the column. This notation intuitively highlights the core advantages of the proposed model/strategy.

### 3.5 Tumor size stratification analysis

To further address the clinical challenge of detecting small lesions (Reviewer #4), we performed a stratified analysis based on tumor diameter: Small (*<* 2 cm), Medium (2 − 5 cm), and Large (*>* 5 cm). As shown in **Table 3**, LCMamba-S demonstrates exceptional robustness in the “Small” category, outperforming the baseline TransResUNet by 2.4% in Dice, validating the effectiveness of the LCAM module in capturing fine-grained details.

**Table 3:** Lesion-size stratified performance on LiTS (Dice score, Mean ± SD).

Method	Small (*<*2 cm)	Medium (2−5 cm)	Large (*>*5 cm)
U-Net	78.45 ± 6.12	89.15 ± 3.20	93.40 ± 2.10
TransResUNet	81.20 ± 5.45	91.30 ± 2.85	94.10 ± 1.85
VM-UNetV2	80.85 ± 5.60	90.95 ± 2.95	93.85 ± 1.95
LCMamba-S	**83.60 ± 5.10**	**92.05 ± 2.65**	**94.45 ± 1.75**

For metrics where higher values indicate superior segmentation accuracy (Dice (%), mIoU (%), Recall (%), Precision (%), F2 (%)), the bold value is the maximum in the column.

For metrics where smaller values reflect more precise boundary delineation (HD95 (mm)), or lower computational cost (Params (M), FLOPs (G), Inference Time), the bold value is the minimum in the column. This notation intuitively highlights the core advantages of the proposed model/strategy.

### 3.6 Computational efficiency analysis

We compared the computational efficiency of our proposed LCMamba variants against state-of-the-art methods in **Table 4**. Benchmarks were conducted on a single NVIDIA RTX A10 GPU (24GB) using FP32 precision with an input resolution of 256 × 256 and a batch size of 1. “Per-Volume” inference time is estimated based on an average volume depth of 150 slices. LCMamba-S achieves a competitive inference speed of 24 ms/slice, significantly faster than Transformer-based counterparts, making it suitable for clinical deployment.

**Table 4:** Computational efficiency benchmark on NVIDIA RTX A10.

Method	Params (M)	FLOPs (G)	Inference time		GPU Mem (MB)
			ms/slice	s/vol	
U-Net	34.5	65.4	18	2.7	1240
Swin-UNETR (3D)	62.8	384.2	N/A	12.5	8400
TransResUNet	48.2	92.1	45	6.8	3100
VM-UNetV2	28.4	54.3	32	4.8	2150
**LCMamba-T (Ours)**	**18.6**	**38.5**	**21**	**3.2**	**1450**
**LCMamba-S (Ours)**	24.2	49.8	24	3.6	1850

For metrics where higher values indicate superior segmentation accuracy (Dice (%), mIoU (%), Recall (%), Precision (%), F2 (%)), the bold value is the maximum in the column.

For metrics where smaller values reflect more precise boundary delineation (HD95 (mm)), or lower computational cost (Params (M), FLOPs (G), Inference Time), the bold value is the minimum in the column. This notation intuitively highlights the core advantages of the proposed model/strategy.

### 3.7 Ablation study

**Figure 3:**
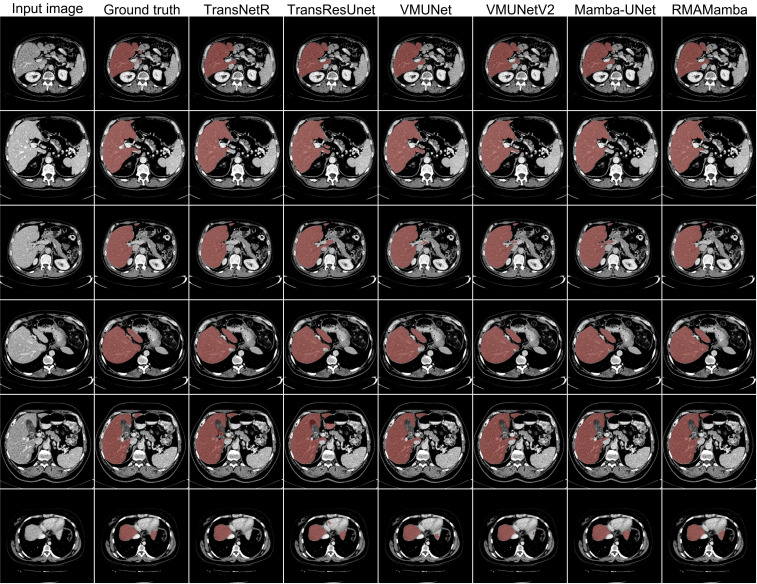
Qualitative results of different methods on the CirrMRI600+ dataset.

**Table 5** presents a comprehensive ablation study verifying the contribution of individual modules. **Figure 3** provides qualitative comparisons for these configurations.

**Table 5:** Ablation study on the CirrMRI 600+ dataset (Mean ± SD).

Exp.	Backbone	Setting	RA	RMA	Dice (%)	mIoU (%)	F2 (%)	HD95
#1	VMamba-Tiny	N=0		x	90.86 ± 3.05	85.63 ± 3.82	91.50 ± 3.55	3.52 ± 1.22
#2	VMamba-Small	N=0		×	91.08 ± 3.00	86.16 ± 3.75	91.09 ± 3.50	3.46 ± 1.18
#5	VMamba-Tiny	N=0			91.17 ± 2.98	86.16 ± 3.65	91.65 ± 3.35	3.50 ± 1.10
#6	VMamba-Small	N=1			**92.08 ± 2.85**	**87.36 ± 3.40**	**92.43 ± 3.20**	**3.39 ± 0.95**

For metrics where higher values indicate superior segmentation accuracy (Dice (%), mIoU (%), Recall (%), Precision (%), F2 (%)), the bold value is the maximum in the column.

For metrics where smaller values reflect more precise boundary delineation (HD95 (mm)), or lower computational cost (Params (M), FLOPs (G), Inference Time), the bold value is the minimum in the column. This notation intuitively highlights the core advantages of the proposed model/strategy.

Furthermore, we conducted an ablation study on augmentation strategies (**Table 6**) and a robustness analysis across different dataset splits (**Table 7**) to ensure the reliability of our proposed method.

**Table 6:** Augmentation strategy ablation on CirrMRI 600+ (Mean ± SD).

Setting	Dice (%)	mIoU (%)	Recall (%)	Precision (%)	F2 (%)	HD95 (mm)
No augmentation	90.91 ± 3.10	85.02 ± 3.90	91.12 ± 3.65	92.04 ± 3.35	91.23 ± 3.50	3.63 ± 1.30
Basic (rot/flip)	91.62 ± 2.95	86.15 ± 3.60	92.40 ± 3.40	92.31 ± 3.20	91.96 ± 3.30	3.51 ± 1.15
Full (ours)	**92.08 ± 2.85**	**87.36 ± 3.40**	**92.96 ± 3.15**	**93.30 ± 3.05**	**92.42 ± 3.20**	**3.39 ± 0.95**

For metrics where higher values indicate superior segmentation accuracy (Dice (%), mIoU (%), Recall (%), Precision (%), F2 (%)), the bold value is the maximum in the column.

For metrics where smaller values reflect more precise boundary delineation (HD95 (mm)), or lower computational cost (Params (M), FLOPs (G), Inference Time), the bold value is the minimum in the column. This notation intuitively highlights the core advantages of the proposed model/strategy.

**Table 7:** Robustness to data splits on CirrMRI 600+ (Mean ± SD).

Model	Protocol	Dice (%)	mIoU (%)	Recall (%)	HD95 (mm)
LCMamba-S	Fixed Split (70/10/20)	91.94 ± 0.24	87.12 ± 0.28	92.81 ± 0.31	3.42 ± 0.06
LCMamba-S	5-fold CV	92.11 ± 0.19	87.29 ± 0.22	92.95 ± 0.27	3.41 ± 0.05

## 4 Conclusion

This paper introduces LCMamba Net, a novel 2D architectural framework that strategically leverages selective state space models to address the computational bottlenecks of high-resolution medical image segmentation. While 3D State-of-the-Art models like nnU-Net? provide global volumetric context, they inherently demand high computational resources and memory, restricting their deployment in real-time or resource-limited clinical environments. Our approach bridges this critical gap, offering competitive accuracy (Dice 92.94%) comparable to 3D baselines while maintaining the high efficiency characteristic of 2D networks. By incorporating the Mamba backbone, LCMamba Net effectively models long-range dependencies within slices, mitigating the limited receptive field issues typical of standard CNNs.

However, a primary limitation of our current slice-wise approach is the lack of inter-slice consistency, as the model does not explicitly learn the Z-axis spatial continuity found in volumetric data. This may result in minor inconsistencies in boundary predictions across sequential slices. Future work will focus on extending the Mamba block to a pseudo-3D or 2.5D framework to capture inter-slice correlations without incurring the full computational cost of 3D convolutions, further enhancing segmentation robustness for clinical applications.

## Data Availability

The original contributions presented in the study are included in the article/supplementary material. Further inquiries can be directed to the corresponding authors.
